# Approval status and evidence for WHO essential medicines for children in the United States, United Kingdom, and Japan: a cross-sectional study

**DOI:** 10.1186/s40545-016-0094-2

**Published:** 2017-01-06

**Authors:** Rumiko Shimazawa, Masayuki Ikeda

**Affiliations:** 1 Department of Clinical Pharmacology, Tokai University School of Medicine, 143 Shimokasuya, Isehara, Kanagawa 259-1193 Japan; 2 Department of Medical Informatics, Kagawa University Hospital, Miki-cho Ikenobe, Kagawa 761-0793 Japan

**Keywords:** Drug approval, Essential medicines list, Neglected infectious diseases, Pharmacopoeias

## Abstract

**Background:**

The WHO Model List of Essential Medicines for Children (EMLc) covers medicines for globally high-burden diseases. Regulatory approval in high-income countries ensures evidence and dosage form but usually focuses on diseases common in those countries and not in low- and middle-income countries.

**Methods:**

This cross-sectional study assessed supporting evidence for the 346 medicines in the 5th WHO EMLc and their approval data from the United States, United Kingdom, and Japan.

**Results:**

Of the 346 EMLc medicines, 307 were approved in one or more of the three countries, 278 of which had supporting evidence of efficacy. The percentage of medicines approved in one or more of the three countries was lowest for antiparasitics (60%) whereas 100% for medicines for cancers and musculoskeletal and respiratory conditions were approved. Five of the 30 EMLc antineoplastics had no supporting paediatric evidence. Of the 39 EMLc medicines unapproved in all three countries, 26 were indicated for neglected infectious diseases (NIDs). Ten of the 26 had supporting paediatric evidence. Seventeen of the 39 unapproved medicines had no paediatric dosage form available, and all 17 were indicated for NIDs.

**Conclusions:**

Most EMLc medicines for diseases common in the three countries had supporting evidence, which was closely associated with approval, whereas a substantial number of medicines for NIDs were unapproved in the three countries, regardless of whether they had supporting evidence. Because of the limited contribution to the EMLc from high income countries, appropriate incentive mechanisms for pharmaceutical companies are required to make paediatric development for NIDs feasible and effective.

**Electronic supplementary material:**

The online version of this article (doi:10.1186/s40545-016-0094-2) contains supplementary material, which is available to authorized users.

## Background

The lack of authorised medicines for children has been an issue of global concern [1–4]. Pharmaceutical companies have been reluctant to invest in developing specific treatments or adapting existing medicines to meet the needs of the paediatric population, mainly because the market is small and therefore of lower commercial interest. The lack of paediatric development raises the ethical concern that children have not benefitted from therapeutic options to the same extent as adults [5, 6].

Age-appropriate dosage forms represent another obstacle in paediatric medication. The absence of an appropriate dosage form for children often results in suboptimal compliance with recommended drug regimens and undermines efficacy or safety [7]. World Health Assembly resolution 60.20 urges member states to take steps to identify age-appropriate paediatric dosage forms that are less expensive to produce, more heat stable, and more convenient to transport than traditional ones [8]. There are few financial incentives, however, for pharmaceutical companies to provide paediatric dosage forms with relevant evidence of efficacy, safety, and tolerability [9].

The World Health Organization (WHO) Model List of Essential Medicines for Children (EMLc) [10] provides a priority list of medicines for paediatric health care needs. Revision and updating of the EMLc by the WHO Expert Committee on the Selection and Use of Essential Medicines [11] requires evaluation of the scientific evidence, based on the comparative effectiveness, safety, cost-effectiveness, and public health need of the medicines. The Committee’s additions to the EMLc are expected to support efforts to reduce the cost of EMLc medicines, as in the case of antiretrovirals [12]. Whereas evidence-based recommendation is essential for regulatory approval in high-income countries (HICs), robust evidence is often unavailable for high-burden conditions such as neglected infectious diseases (NIDs) [13] prevalent in low- and middle-income countries (LMICs). Reviewing applications for the EMLc is challenging because the Committee must often make decisions to ensure access to indispensable treatment in the absence of evidence. This is the reason WHO uses the Grading of Recommendations Assessment, Development and Evaluation (GRADE) approach [14] to make recommendations for medicine use in areas where there is a lack of evidence.

Compared with reviews in EMLc, clinical trials to provide evidence are essential for regulatory approval of medicines for children in HICs. Development and regulation of medicines are coordinated among International Conference on Harmonisation of Technical Requirements for Registration of Pharmaceuticals for Human Use (ICH) countries, all of which are HICs. The ICH Tripartite Guideline [15] stipulates conditions for paediatric medicinal product development. ICH regulatory authorities, the United States Food and Drug Administration (FDA), the European Medicines Agency, and the Pharmaceutical and Medical Devices Agency (PMDA), all review the clinical trial data. Considerable attention is paid to internal validity, safety, efficacy, and manufacturing, and focus on the risk-benefit profile of a product. Subsequent drug approval is independent of the public health perspective and the cost of the medicine.

HICs have not directly improved access to the EMLc medicines [1] and may even exploit children in LMICs [16, 17] to establish evidence. For example, in a clinical trial involving premature babies with respiratory distress syndrome that was led by a company from the United States (US), some participants were to be given a placebo despite the availability of drugs for the condition [17]. However, HICs can contribute to the EMLc in cooperation with the WHO. The paediatric medicine initiatives of HICs [18] have established and expanded networks with specific expertise in performing clinical trials and have improved the scientific evidence and availability of paediatric dosage forms. These data have helped the Committee [11] to review and update the EMLc.

The aim of the present study was to investigate the implications of approval in HICs and supporting evidence of efficacy for the EMLc medicines. For this purpose, we analysed the evidence and approval status of EMLc medicines in the US, United Kingdom (UK), and Japan, which are all members of the ICH.

## Methods

### Definition of approval status and analysis of approved products

This study included medicines appearing on the 5th EMLc [10]. We identified labels available in July 2015 from the DailyMed [19] and Drugs@FDA websites [20] for US Structured Product Labels, the electronic Medicines Compendium [21] and the Medicines and Healthcare Products Regulatory Agency websites [22] for UK Summaries of Product Characteristics, and the website of the PMDA [23] for Japanese labels. We selected these countries for our comparison because of the similarities in their drug regulations, as all three are members of the ICH. The UK provides a good comparison with the US and Japan because it shares a common language with the US and provides universal pharmaceutical coverage, as does Japan.

When the product label covered the indication, dosage, and route of administration for children at any age, the label was considered to be approved for paediatric use [24]. If a medicine had been discontinued as a prescription drug but was available as an over-the-counter drug for children, we designated it as approved. In addition to the paediatric approval status, we also checked the dosage form on the label to determine whether it covered any of the paediatric dosage forms on the WHO list. When we found any paediatric dosage form available in at least one of the three countries, we considered the paediatric dosage form to be available. To enable data from the current study to be used for comparison in future studies, we analysed the data according to the Anatomical Therapeutic Chemical (ATC) Classification System [25], which classifies drugs by the organ or system on which they act and their therapeutic, pharmacological, and chemical properties.

### Definition of supporting evidence and analysis of evidence

To evaluate the supporting evidence for each medicine, we used the DRUGDEX® System (Truven Health Analytics, Ann Arbor, MI, USA) [26], which is recognised by the Centers for Medicare and Medicaid Services as a pharmaceutical compendium that describes the efficacy and scientific documentation for prescription drugs and has been used to approve payment. When the efficacy of a medicine was described as “Effective” or “Evidence favors efficacy” on DRUGDEX, we considered that supporting evidence was available for the indication. When the therapeutic use was indicated as “See Drug Consult reference” and the dosage and route of administration for children were specified in the reference, we considered that supporting evidence was available for the medicine. When the efficacy of a medicine was described as “Evidence is inconclusive” or “Ineffective”, we considered that supporting evidence was not available for the indication. When a medicine or its evidence was not listed on DRUGDEX, we designated it as “No evidence”.

## Results

There were a total of 346 medicines on the 5th EMLc (see Additional file 1). Table 1 shows that approval status in the three countries is closely related to evidence of efficacy. Supporting evidence was provided for 287 (91%) of the 307 medicines approved in the US, UK or Japan whereas evidence was available for only 13 (33%) of the 39 medicines unapproved in in the three countries.

**Table 1:** Relationship between approval status and evidence of essential medicines for children

Paediatric approval	Total	Evidence				Supporting evidence rate
		Supporting	Inconclusive	Ineffective	No evidence	
US/UK/Japan	160	155	2	0	3	97%
Two of the three	105	96	0	0	9	91%
One of the three	42	27	2	1	12	64%
None	39	13	4	0	22	33%
Total	346	291	8	1	46	84%

There were 29 medicines approved in one or more of the three countries without supporting paediatric evidence. These medicines are classified in Table 2 according to supporting evidence in children and adults. Of the 29 medicines, 26 had supporting evidence in adults.

**Table 2:** Number of essential medicines for children approved without paediatric evidence in the US, UK, or Japan, in groups classified according to supporting evidence in children or adults

		Evidence in adults			
		Supporting	Inconclusive	No evidence	Total number
Evidence in children	Inconclusive	3	0	1	4
	Ineffective	0	0	1	1
	No evidence	23	0	1	24
	Total number	26	0	3	29

In Japan, 162 (47%) of the 346 EMLc medicines were unapproved for children whereas 78 (22%) and 66 (19%) were unapproved in the US and UK, respectively (Table 3). Less than half of the 346 EMLc medicines (160; 46%) were approved in all three countries whereas 307 (89%) were approved in at least one of the three countries.

**Table 3:** Approval status of essential medicines for children in the US, UK, and Japan

Approval status	US	(%)	UK	(%)	Japan	(%)
Approved for both adults and children	261	(75)	275	(79)	180	(52)
Approved for children only	7	(2.0)	5	(1.4)	4	(1.2)
Unapproved for children	42	(12)	15	(4.3)	87	(25)
Unapproved for both adults and children	36	(10)	51	(15)	75	(22)

Table 4 shows the number of medicines according to approval status and ATC code. Anti-infectives (code J) were most represented (99/346; 29%), followed by antiparasitics (code P) (40/346; 12%). Antiretrovirals included in anti-infectives and itemized in Additional file 1 (Nos.113–126) were all approved in one or more of the three countries. The percentage of medicines approved in one or more of the three countries was lowest for antiparasitics (24/40; 60%) whereas it was 100% for antineoplastics and medicines for musculoskeletal and respiratory systems. Of the 30 antineoplastics listed in Table 4 and itemized in Additional file 1 (Nos.168–197), only eight, including three corticosteroids and two supportive care agents, were categorised as effective whereas five cytotoxic agents (bleomycin, cisplatin, etoposide, ifosfamide, and vinblastine) were categorised as no evidence.

**Table 4:** Number of essential medicines for children according to approval status, evidence, and Anatomical Therapeutic Chemical (ATC) code

ATC code^a^	A	B	C	D	H	J	L	M	N	P	R	S	V	Total
Approved^b^	31	19	11	23	19	99	30	6	32	40	8	11	17	346
Unapproved^c^	1	1	1	1	2	10	0	0	3	16	0	1	3	39
Rate of unapproved (%)	3.2	5.3	9.1	4.3	11	10	0	0	9.4	40	0	9.1	18	11

^a^Anatomical Therapeutic Chemical (ATC) classification codes. A, alimentary tract and metabolism; B, blood and blood-forming organs; C, cardiovascular system; D, dermatologicals; G, genitourinary system and sex hormones; H, systemic hormonal preparations, excluding sex hormones; J, general anti-infectives for systemic use; L, antineoplastic and immunomodulating agents; M, musculoskeletal system; N, nervous system; P, antiparasitic products; R, respiratory system; S, sensory organs; V, various

^b^Essential medicines for children approved in the US, UK, or Japan

^c^Essential medicines for children unapproved in the US, UK, and Japan

There were 39 medicines unapproved for use in children in all three countries. Of these, 26 were indicated for NIDs. Table 5 shows the 39 medicines classified according to supporting evidence in children or adults. Of these, there was supporting paediatric evidence for 13 (Table 6) but inconclusive or no evidence for 26 medicines. Of the 13 with supporting paediatric evidence but unapproved in the three countries, 10 were indicated for NIDs (Table 6). Of the 26 without paediatric evidence, there was supporting evidence for use in adults for 17 medicines (Table 7). The therapeutic uses for these 17 include NIDs and rare diseases in children, e.g., coagulation factor deficiency, mydriasis induction in cataract surgery, and ethylene glycol toxicity. Nine medicines unapproved in all three countries had no supporting evidence in either adults or children (Table 8). Eight of these nine medicines, excluding morphine for sedation, were indicated for NIDs.

**Table 5:** Number of essential medicines for children unapproved in the US, UK, and Japan, classified according to supporting evidence in children or adults

		Evidence in adults			
		Supporting	Inconclusive	No evidence	Total number
Evidence in children	Supporting	12	1	0	13
	Inconclusive	3	1	0	4
	No evidence	14	3	5	22
	Total number	29	5	5	39

**Table 6:** Essential medicines for children unapproved in the US, UK, and Japan with paediatric evidence

Name	ATC^a^	Therapeutic use	Adult efficacy^b^	Paediatric efficacy^b^	Dosage form^c^
Potassium iodide	D	Cutaneous sporotrichosis	Favours efficacy	Favours efficacy	A
Doxycycline	J	Malaria	Favours efficacy	Favours efficacy	A
Amikacin	J	Tuberculosis	Favours efficacy	Favours efficacy	A
Rifampicin	J	Leprosy	Favours efficacy	Favours efficacy	A
Capreomycin	J	Tuberculosis	Effective	Favours efficacy	A
Diphtheria antitoxin	J	Diphtheria	Effective	Effective	NA
Isoflurane	N	General anaesthesia	Effective	Effective	A
Primaquine	P	Malaria	Favours efficacy	Favours efficacy	A
Artesunate	P	Malaria	Effective	Effective	NA
Artesunate + Mefloquine	P	Malaria	Effective	Effective	NA
Artesunate + Amodiaquine	P	Malaria	Effective	Effective	NA
Benznidazole	P	American trypanosomiasis	Inconclusive	Favours efficacy	NA
Protamine sulfate	V	Heparin overdose	Effective	Favours efficacy	A

^a^Anatomical Therapeutic Chemical (ATC) classification codes: D, dermatologicals; J, general anti-infectives for systemic use; L, antineoplastic and immunomodulating agents; N, nervous system; P, antiparasitic products; V, various

^b^Adult or paediatric efficacy: Inconclusive, evidence is inconclusive; Favours efficacy, evidence favours efficacy

^c^Dosage Form: A, available; NA, not available

**Table 7:** Essential medicines for children unapproved in the US, UK, and Japan with supporting evidence in adults but not in children

Name	ATC^a^	Therapeutic use	Adult efficacy^b^	Paediatric efficacy^b^	Dosage form^c^
Fresh-frozen plasma	B	Coagulation factor deficiency	Effective	No evidence	A
Dopamine	C	Heart failure	Effective	No evidence	A
Fludrocortisone	H	Adrenal insufficiency	Effective	No evidence	A
Lugol’s solution	H	Thyroid storm	Effective	No evidence	A
Levofloxacin	J	Tuberculosis	Favours efficacy	No evidence	A
Amphotericin B	J	Leishmaniasis	Effective	No evidence	A
Clofazimine	J	Leprosy	Effective	No evidence	A
Ribavirin	J	Viral haemorrhagic fevers	Favours efficacy	No evidence	A
Amitriptyline	N	Pain in palliative care	Effective	Inconclusive	A
Sodium stibogluconate	P	Leishmaniasis	Favours efficacy	No evidence	NA
Melarsoprol	P	African trypanosomiasis	Favours efficacy	No evidence	NA
Suramin sodium	P	African trypanosomiasis	Favours efficacy	No evidence	NA
Eflornithine	P	African trypanosomiasis	Effective	No evidence	NA
Triclabendazole	P	Infection by Paragonimus	Favours efficacy	Inconclusive	NA
Epinephrine (Adrenaline)	S	Mydriasis induction in cataract surgery	Favours efficacy	No evidence	A
Fomepizole	V	Ethylene glycol toxicity	Effective	Inconclusive	A
Mesna	V	Prophylaxis of ifosfamide-induced haemorrhagic cystitis	Effective	No evidence	A

^a^Anatomical Therapeutic Chemical (ATC) classification code: B, blood and blood-forming organs; C, cardiovascular system; H, systemic hormonal preparations, excluding sex hormones; J, general anti-infectives for systemic use; N, nervous system; P, antiparasitic products; S, sensory organs; V, various

^b^Adult or paediatric efficacy: Inconclusive, evidence is inconclusive; Favours efficacy, evidence favours efficacy

^c^Dosage Form: A, available; NA, not available

**Table 8:** Essential medicines for children unapproved in the US, UK, and Japan without supporting evidence either in adults or children

Name	ATC^a^	Therapeutic use	Adult efficacy^b^	Paediatric efficacy^b^	Dosage Form^c^
Paromomycin	A	Leishmaniasis	No evidence	No evidence	NA
Linezolid	J	Tuberculosis	No evidence	No evidence	A
Morphine	N	Sedation^d^	No evidence	No evidence	A
Amodiaquine	P	Malaria	No evidence	No evidence	NA
Artemether	P	Malaria	Inconclusive	Inconclusive	NA
Nifurtimox	P	African trypanosomiasis	Inconclusive	No evidence	NA
Nifurtimox	P	American trypanosomiasis	Inconclusive	No evidence	NA
Levamisole	P	Helminth infection	Inconclusive	No evidence	NA
Benzyl benzoate	P	Scabies	No evidence	No evidence	NA

^a^Anatomical Therapeutic Chemical (ATC) classification code: A, alimentary tract and metabolism; J, general anti-infectives for systemic use; N, nervous system; P, antiparasitic products

^b^Adult or paediatric efficacy: Inconclusive, evidence is inconclusive

^c^Dosage Form: A, available; NA, not available

^d^Morphine is listed in the EMLc as the drug of choice for the treatment of severe acute or chronic pain, which was acknowledged as approved in the three countries. However, efficacy of morphine for sedation has not been established or approved in any of the three countries. That is why the listing of morphine indicated to sedation as unapproved

Regarding the availability of a paediatric dosage form for the 39 medicines unapproved for children in all three countries, 17 had no paediatric dosage form (Table 9). All 17 of these were indicated for NIDs. Of these 17 medicines, 12 had no supporting evidence in children.

**Table 9:** Essential medicines for children unapproved in the US, UK, and Japan without a paediatric dosage form

Name	ATC^a^	Therapeutic use	Adult efficacy^b^	Paediatric efficacy^b^
Paromomycin	A	Leishmaniasis	No evidence	No evidence
Diphtheria antitoxin	J	Diphtheria	Effective	Effective
Amodiaquine	P	Malaria	No evidence	No evidence
Artemether	P	Malaria	Inconclusive	Inconclusive
Artesunate	P	Malaria	Effective	Effective
Artesunate + Mefloquine	P	Malaria	Effective	Effective
Artesunate + Amodiaquine	P	Malaria	Effective	Effective
Benznidazole	P	American trypanosomiasis	Inconclusive	Favours efficacy
Sodium stibogluconate	P	Leishmaniasis	Favours efficacy	No evidence
Nifurtimox	P	African trypanosomiasis	Inconclusive	No evidence
Nifurtimox	P	American trypanosomiasis	Inconclusive	No evidence
Melarsoprol	P	African trypanosomiasis	Favours efficacy	No evidence
Suramin sodium	P	African trypanosomiasis	Favours efficacy	No evidence
Eflornithine	P	African trypanosomiasis	Effective	No evidence
Triclabendazole	P	Infection by Paragonimus	Favours efficacy	Inconclusive
Levamisole	P	Helminth infection	Inconclusive	No evidence
Benzyl benzoate	P	Scabies	No evidence	No evidence

^a^Anatomical Therapeutic Chemical (ATC) classification code: A, alimentary tract and metabolism; J, general anti-infectives for systemic use; P, antiparasitic products

^b^ Adult or paediatric efficacy: Inconclusive, evidence is inconclusive; Favours efficacy, evidence favours efficacy

## Discussion

This study revealed that most of the EMLc medicines for diseases common in HICs had supporting evidence that was closely associated with approval in the three countries whereas medicines for diseases prevalent in LMICs but not in HICs were on the list as essential even though they were unapproved in the three countries or had no supporting evidence.

Although regulation of medicines is basically harmonised among the US, UK, and Japan, which are all members of the ICH, we found that the approval gaps between the US and Japan and between the UK and Japan were much larger than that between the US and the UK. Another study reported similar gaps between the US and Japan in medicines for adults [27]. The unique requirement of domestic dose-finding studies [28] has likely resulted in delays in filing applications for new medicines of non-Japanese origin, which subsequently created the gap for paediatric approval in Japan. Extrapolation of adult data for paediatric approval, if possible, would help to close this gap. However, the ICH E11 guideline for clinical trials in the paediatric population [15] stipulates that pharmacokinetic studies should generally be performed to support development of dosage forms and determine pharmacokinetic parameters in different age groups, so as to support dosing recommendations.

Paediatric medicine initiatives in the US and European Union (EU) have aimed to improve the availability of medicines for children [18]. In contrast with the US and EU, no comprehensive legislation exists in Japan to provide incentives and facilitate paediatric development. Although Japan has enhanced its contribution to international harmonisation through the ICH and has followed the US and EU by enacting its own paediatric medicine initiatives, the progress of these regulatory reforms has been modest [18, 29].

The approval status of EMLc medicines in the three countries varied depending upon therapeutic area. Medicines for diseases common in HICs, e.g., HIV infection, respiratory diseases and cancers, were all approved in one or more of the three countries. Although all 30 antineoplastics were approved, their evidence was not always robust; five cytotoxic agents were categorised as having no evidence. This means that when reviewing antineoplastics, regulatory authorities in these three countries might give priority to effectiveness rather than to efficacy. The recent proposal of the WHO Expert Committee’s 2015 meeting with respect to antineoplastics [12] opened up the doors to rigorous evidence requirements for other categories of medicines.

Among the 39 medicines unapproved in all three countries studied, there were differences in the gradient of supporting evidence and in the availability of a paediatric dosage form. Although evidence is essential for regulatory approval in HICs, medicines with good evidence do not always receive approval. Of the 39 medicines, 13 with supporting evidence were for diseases rare among children in HICs; 10 of the 13 were for NIDs. Because of the small market and low financial interest, there are few financial incentives for pharmaceutical companies to seek approval for the 13 medicines with supporting evidence in children. Although paediatric dosage forms are unavailable in the US, UK, and Japan for five of these 13 medicines, appropriate dosage forms may be available in another country or could be developed via options other than full development [30, 31].

Of the other 26 medicines unapproved in all three countries without supporting paediatric evidence, 17 had evidence in adults. The lack of paediatric evidence likely results from limited numbers of study participants, probably because the prevalence of most indications for the 17 medicines is very low in HICs. The need for separate paediatric development may be reduced by extrapolating prior knowledge acquired during the development of these medicines for adults, on the basis of assuming a similar disease for the proposed paediatric indication [31, 32]. The remaining nine of the 26 unapproved medicines have no evidence in either adults or children. Eight of these nine, excluding morphine for sedation, are antiparasitic or anti-infective products indicated for NIDs.

The distribution of EMLc medicines according to therapeutic area shows that the EMLc addresses the most pressing public health concerns of children around the world, namely, NIDs [13]. Our study showed that 26 of the 39 medicines unapproved in all three countries were indicated for NIDs. Whereas all of the antiretrovirals listed on the EMLc were approved in one or more of the three countries assessed, we found that 40% of antiparasitics were unapproved in all three countries despite their public health relevance in LMICs. This distinction highlights the interest of HICs regarding infectious diseases that are prevalent in their own countries but not in LMICs.

Pharmaceutical companies, in association with researchers, are the key stakeholders in paediatric development. Incentives are imperative for them to develop medicines for NIDs [33, 34] because the market potential is quite low and external funding has been reduced [35]. A combination of push and pull incentive mechanisms [34] has been proposed as being suitable to promote clinical development for NIDs. Push mechanisms, such as research grants or publicly financed institutions, support basic research whereas pull mechanisms, such as priority review vouchers or extension of the medicine’s exclusivity period [33], have the potential to stimulate research and development of medicines for NIDs. Because of the limited resources in LMICs, these incentives should be applied to paediatric development for NIDs of high priority, which are the WHO roadmap targets for eradication and elimination [36].

Several limitations of this analysis should be noted. First, we used DRUGDEX as the only source, to classify supporting evidence of efficacy. Whereas this database is well recognised and is easily available, it is compiled by an expert working group in an HIC, namely, the US. By defining the presence or absence of supporting evidence according to DRUGDEX, the evidence we identified as “no evidence” may include varying degrees of scientific evidence that fell short of our definition. This limitation would be particularly applicable to medicines for NIDs. Second, because we had to rely on data collected for different purposes by other groups, we were limited in our ability to capture the gradient of evidence for EMLc medicines. Third, we could not address social obstacles to accessing EMLc medicines such as cost, intellectual property, or logistics, because we focused on the scientific aspects of the EMLc. Finally, we were limited to collecting approval data from the US, UK, and Japan and omitted approvals and development outside these regions.

## Conclusion

HICs have made a substantial contribution to improve the scientific evidence and availability of paediatric dosage forms for EMLc in some therapeutic areas, such as antiretrovirals and antineoplastics. To date, however, there has been little commitment of HICs to NIDs given their large economies. Deficiencies in the ability to meet the critical needs of children worldwide remain, particularly with respect to NIDs. To make paediatric development for NIDs feasible and effective, appropriate incentive mechanisms for pharmaceutical companies should be applied towards the eradication and elimination of NIDs.

## Additional file

Additional file 1: Approval status in the United States, United Kingdom, and Japan and supporting evidence for medicines on the 5th WHO Model List of Essential Medicines for Children. (XLSX 33 kb)

## Abbreviations

ATC: Anatomical therapeutic chemical classification system
EMLc: WHO model list of essential medicines for children
EU: European Union
FDA: United States Food and Drug Administration
GRADE: Grading of Recommendations Assessment, Development and Evaluation
HICs: High-income countries
ICH: International conference on harmonisation of technical requirements for registration of pharmaceuticals for human use
LMICs: Low- and middle-income countries
NIDs: Neglected infectious diseases
PMDA: Pharmaceutical and Medical Devices Agency
UK: United Kingdom
US: United States
WHO: World Health Organization
